# EXPRESSIONS OF INTERSECTIONALITY IN OLD AGE THROUGH NARRATIVE RESEARCH AND PHOTO EXHIBITION

**DOI:** 10.1093/geroni/igae098.1137

**Published:** 2024-12-31

**Authors:** Ksenya Shulyaev, Tova Band-Winterstein, Tal Dekel, Zvi Eisikovits, Reem Nashef-Hamuda

**Affiliations:** University of Haifa, Haifa, Hefa, Israel; University of Haifa, Haifa, HaZafon, Israel; Kibbutzim College of Education, Tel Aviv, HaMerkaz, Israel; University of Haifa, Haifa, Hefa, Israel; Haifa University, Haifa, HaZafon, Israel

## Abstract

This presentation analyzes the complex experiences of old women navigating multiple marginalized identities, through an intersectional lens. There is relatively limited in-depth representation of older women at the crossroads of multiple social disadvantages. This project addresses this gap through a collaboration with 15 older women in Israel from diverse minority groups (lesbian/transgender, women with disabilities, Israeli Arabs, immigrants from Ethiopia, and former USSR). The project aims to visualize older women’s self from an intersectional perspective by creating a visual documentary – (a photo exposition) with added verbal narrative of their life stories. Utilizing visual narratives and in-depth interviews, the project explores the unique experiences of old women shaped by age and additional marginalized identities. Professional artists guided the photo sessions, ensuring the women’s vision and representation were prioritized. The resulting artwork was exhibited publicly at the University of Haifa library with accompanying small group sessions aimed at increasing awareness of students and the community about discrimination against older women. Visitors to the exhibition will be interviewed for further analysis and understanding of the impact of the exhibition in terms of visibility and attitudinal change. The exhibition will become a permanent online display on the Minerva Center of Intersectionality in Aging website. The presentation of on GSA conference includes photos and citations of the life stories of women who participated in the project. This unique project illuminates the lived reality of aging women intersectionality, offering insights to gerontological researchers and practitioners, challenges stereotypes and empowers women to express their experiences visually

